# A World Café Approach to Exploring Perspectives on Diabetes Stigma in the United Kingdom

**DOI:** 10.1111/hex.70023

**Published:** 2024-09-06

**Authors:** Amy Elizabeth Burton, Alison Owen, Jennifer Taylor, Sarah Elizabeth Dean, Rachel Povey

**Affiliations:** ^1^ Centre for Applied Psychology and Performance Staffordshire University Stoke‐on‐Trent UK

**Keywords:** diabetes, management, policy, stigma, World Café

## Abstract

**Introduction:**

Research has found that a high percentage of people with diabetes experience stigma, with perceptions of stigma being significantly higher among people with Type 1 diabetes compared to those with Type 2 diabetes. These experiences of diabetes stigma can lead to psychological, behavioural and medical consequences. The aim of the current study was to explore the perceptions of diabetes stigma and propose strategies for addressing this from the perspective of key stakeholders.

**Methods:**

A mixed methods design was used, commencing with an online qualitative survey (*n* = 128) and followed by a World Café (*n* = 11), where attendees reflected on their own experiences with stigma and discussed the survey findings.

**Results:**

The survey indicated that 75% of those with Type 1 diabetes and 51% with Type 2 had experienced diabetes stigma. The World Café identified three main areas that participants felt impacted their experiences with stigma or had the potential to help improve stigma: healthcare interactions, public awareness and media representations.

**Conclusions:**

The findings supported previous research showing that diabetes stigma is prevalent among people with diabetes. The World Café was an excellent means of sharing knowledge and experiences among stakeholders, the findings of which will inform strategies to bring about change.

**Patient or Public Contribution:**

World Café is a collaborative method where stakeholders contribute to the production and analysis of data through rounds of discussion and feedback.

## 1 Introduction

Diabetes mellitus is a common chronic metabolic disease experienced by approximately 529 million people worldwide [1] and 4.4 million people within the United Kingdom [2]. Diabetes results in hyperglycaemia; in around 10% of diabetes cases this is caused by poor insulin production by the pancreas (Type 1) and in around 90% of cases result from insulin resistance or dysfunction (Type 2) [3].

Stigma in diabetes is defined as ‘negative attitudes, judgement, discrimination or prejudice against someone because of their diabetes’ [4]. A survey of 12,000 people with Type 1 and Type 2 diabetes illustrated that experiencing stigma was common and that stigma experiences were significantly higher among respondents with Type 1 diabetes than among those with Type 2 [5]. There are different sources of diabetes stigma, for example, people with Type 1 diabetes may experience stigma due to the association of injections and illicit drug use [6, 7], whereas those with Type 2 diabetes may experience stigma due to the belief by others that it is caused by individual lifestyle factors [8, 9]. Diabetes stigma can lead to psychological, behavioural and medical consequences [10], so it is essential to explore this topic with the people who experience it, and through working with them, develop strategies to help mitigate it.

Participatory research methods facilitate the co‐construction of research and knowledge in collaboration with those affected by an issue of interest [11]. Through partnership working, participatory approaches can facilitate quality research, empowerment, capacity building and new activities that enable positive change and improved outcomes within a range of health contexts [12]. World Café is a participatory tool that motivates participant stakeholders to bring about change through discussion activities and shared dialogue [13, 14]. This approach facilitates co‐produced knowledge and identification of issues of importance and potential solutions [13]. World Café has previously been used to explore stakeholder perspectives on several health‐related issues [15, 16, 17] and to better understand issues and concerns related to stigma for the police service [18] and homeless young people [19]. To our knowledge, World Café has not been used to explore diabetes stigma. Therefore, we sought to explore the perceptions of diabetes stigma and strategies for addressing this from the perspective of key stakeholders using the World Café.

## 2 Materials and Methods

### 2.1 Design

The research employed a convergent mixed methods design whereby both qualitative and quantitative data were collected and analysed within a similar time frame, with one element of data collection used to inform the other [20]. Data were initially collected through an online survey that included both quantitative and qualitative surveys, and the results of this survey informed a World Café [13].

### 2.2 Subjects

Adverts for a survey and a free diabetes event were shared on social media, through charities and via several mailing lists. This was open to stakeholders with an interest in diabetes stigma, including people living with diabetes, their family members or carers and healthcare or other professionals. A self‐selected convenience sample followed a Qualtrics [21] link to the information sheet, consent form and survey. At the end of the survey, expressions of interest for attending an in‐person World Café were sought.

A total of 128 participants completed the survey representing a large sample for qualitative survey research [22]. Of these, 20 expressed an interest in attending the World Café, and 13 completed a second informed consent process to register. On the day, 10 attended the World Café, and one attended with their daughter who also completed a consent form. Therefore, 11 participants took part in the World Café (Type 2 diabetes: *n* = 7; Type 1 diabetes: *n* = 1; carer/family member of someone with Type 1: *n* = 2; healthcare professional [HCP]: *n* = 1). This represents a medium‐sized sample for an approach using interactive qualitative data collection methods [22]. Full demographic details are provided in Table 1.

**Table 1:** Survey respondent demographics by group.

	Total	Type 1	Type 2	Parent	HCP	Carer	Other	World Cafe
Sample size	128	32	59	10	8	7	12	11
Experienced diabetes stigma?								
Yes	67 (54%)	24 (75%)	30 (51%)	7 (70%)	1 (12%)	3 (43%)	1 (8%)	8 (73%)
No	51 (41%)	7 (22%)	26 (44%)	2 (20%)	6 (75%)	1 (14%)	10 (83%	2 (18%)
No response	10 (8%)	1 (3%)	3 (5%)	1 (10%)	1 (12%)	3 (43%)	1 (8%)	1 (9%)
Age; number (%)								
18–30	16 (13%)	7 (22%)	3 (5%)	—	—	—	3 (38%)	—
31–40	26 (20%)	7 (22%)	3 (5%)	50 (50%)	3 (38%)	1 (14%)	4 (31%)	3 (27%)
41–50	23 (18%)	4 (13%)	9 (15%)	40 (40%)	4 (50%)	4 (57%)	1 (8%)	1 (9%)
51–60	32 (25%)	8 (25%)	21 (36%)	1 (10%)	1 (13%)	1 (14%)	—	3 (27%)
61–70	15 (12%)	4 (13%)	10 (17%)	—	—	1 (14%)	2 (15%)	2 (18%)
70+	16 (13%)	2 (6%)	13 (22%)	—	—	—	1 (8%)	1 (9%)
Unknown	—	—	—	—	—	—	—	1 (9%)
Gender								
Male	37 (29%)	11 (35%)	22 (37%)	1 (10%)	1 (13%)	—	3 (23%)	3 (27%)
Female	90 (70%)	20 (63%)	37 (63%)	9 (90%)	7 (87%)	7 (100%)	10 (77%)	8 (73%)
Not reported	1 (< 1%)	1 (3%)	—	—	—	—	—	—
Ethnicity; number (%)								
White: English/Welsh/Scottish/Northern Irish/British	110 (86%)	32 (100%)	51 (86%)	7 (70%)	4 (50%)	6 (86%)	11 (85%)	9 (2%)
White Irish	1 (< 1%)	—	1 (2%)	—	—	—	—	—
Any other White background	1 (2%)	—	—	1 (10%)	2 (25%)	—	—	—
Asian/Asian British: Chinese	1 (< 1%)	—	—	—	1 (13%)	—	—	—
Asian/Asian British: Pakistani	3 (2%)	—	2 (3%)	—	—	1 (14%)	—	2 (18%)
Black/Black British: African	2 (2%)	—	2 (3%)	—	—	—	—	—
Black/Black British: Caribbean	1 (< 1%)						1 (8%)	—
Any other mixed/multiple background	1 (< 1%)	—	—	1 (10%)	—	—	—	—
Any other background	3 (2%)	—	2 (3%)	—	—	—	1 (8%)	—
Not reported	3 (2%)	—	1 (2%)	1 (10%)	1 (13%)	—	—	—
Education								
School leaver before 16	6 (5%)	1 (3%)	4 (7%)	—	—	—	1 (8%)	—
School leaver at 16	9 (7%)	6 (19%)	2 (3%)	1 (5%)	—	—	—	—
Further education	32 (25%)	8 (25%)	10 (17%)	5 (50%)	1 (13%)	4 (57%)	4 (31%)	1 (9%)
Higher education	46 (36%)	8 (25%)	28 (47%)	2 (20%)	1 (13%)	2 (29%)	5 (38%)	5 (45%)
Postgraduate education	34 (27%)	9 (28%)	14 (24%)	2 (20%)	6 (75%)	1 (14%)	3 (23%)	4 (36%)
Prefer not to say/not reported	1 (< 1%)	—	1 (2%)	—	—	—	—	1 (9%)
Employment								
Paid employment: full‐time	48 (38%)	15 (47%)	16 (27%)	5 (50%)	6 (75%)	5 (57%)	2 (15%)	2 (18%)
Paid employment: part‐time	25 (20%)	6 (19%)	7 (12%)	2 (20%)	—	3 (43%)	8 (62%)	2 (18%)
Retired	33 (26%)	7 (22%)	25 (42%)	—	—	—	1 (8%)	3 (27%)
Self‐employed	2 (2%)	—	2 (3%)	—	—	—	—	1 (9%)
Voluntary	3 (2%)	—	3 (5%)	—	—	—	—	1 (9%)
Seeking work	3 (2%)	2 (6%)	1 (2%)	—	—	—	—	—
Carer	3 (2%)	—	1 (2%)	1 (10%)	—	—	—	—
Student	4 (3%)	—	—	—	2 (25%)	—	—	—
Other	6 (5%)	2 (6%)	4 (7%)	—	—	—	—	—
Prefer not to say/Not reported	1 (< 1%)	—	—	1 (10%)	—	—	—	1 (9%)
Location								
UK	125 (98%)	32 (100%)	58 (98%)	10 (100%)	7 (87%)	7 (100%)	12 (92%)	11 (100%)
USA	1 (< 1%)	—	—	—	1 (13%)	—	—	—
Not reported	2 (2%)	—	1 (2%)	—	—	—	1 (8%)	—

### 2.3 Data Collection

#### 2.3.1 Initial Survey

The survey was used to inform the discussions to be held at the World Café event and, therefore, explored issues related to the topic of interest. This included demographic data and questions about diabetes status or interest. In addition, participants defined diabetes stigma, identified whether they had experienced diabetes stigma, explained their own or observed experiences of diabetes stigma, listed their top three priorities for addressing diabetes stigma and described any diabetes stigma interventions they were aware of.

#### 2.3.2 World Café Event Structure

The World Café was held at Staffordshire University in July 2023. The facilitators were health psychologists with diabetes research experience. The agenda and process are outlined in Table 2. An opening presentation overviewed the research definition of stigma, research findings from previous publications by the facilitators and headline findings from the survey. The group discussions addressed three key questions facilitated by reflection on attendees' own experiences and the survey findings: (1) ‘What are your thoughts about and experiences of diabetes stigma?’; (2) ‘What are the biggest barriers to addressing diabetes stigma?’; (3) ‘What can be done to reduce diabetes stigma?’.

**Table 2:** An outline of the World Cafe process and associated World Cafe principles.

Activity	Description	World Café principle [13]
Welcome and introductions	Participants were welcomed to the session, and what was going to happen throughout the day was explained. This included an ice‐breaker activity during which participants selected a picture card that represented their experience with diabetes/why they were attending the event. Participants introduced themselves by discussing their choice of card.	1. Setting the context/ 2. Creating a hospitable space.
Presentation about diabetes stigma and the results of the pre‐event survey	The lead facilitators delivered a presentation to participants using PowerPoint slides, talking about their interest and previous research into diabetes stigma and outlining the key findings of the pre‐event survey.	1. Setting the context
Lunch and World Café seating	Participants helped themselves to a buffet lunch and were asked to sit on one of two tables with two table‐hosts on each table, post‐its, poster paper, flipchart paper and a laptop set up with a Teams meeting to record discussion.	2. Creating a hospitable space
Question 1 of World Café	We asked: What are your thoughts about and experiences of diabetes stigma?	3. Explore questions that matter
Rotation	We allowed the self‐selection of participants to move to a different group. Table hosts welcomed newcomers.	4. Encourage everyone's contribution
Summary of key findings	Table host reported the main points of Question 1 to the new group members and newcomers reported the main points from their previous groups' discussion. These were then fed back to the event facilitator and recorded on poster paper.	5. Cross‐pollinate and connect diverse perspectives 7. Harvest and share collective discoveries
Question 2 of World Café	We asked: What are the biggest barriers to addressing diabetes stigma?	3. Explore questions that matter
Rotation	Again, we allowed self‐selection of participants to move to a different group. Table hosts welcomed newcomers.	4. Encourage everyone's contribution
Summary of key findings	Table host reported the main points of Question 2 back to the group, and newcomers reported the main points from their previous groups' discussion. Ideas were again fed back to the event facilitator and recorded on poster paper.	5. Cross‐pollinate and connect diverse perspectives 7. Harvest and share collective discoveries
Question 3 of World Café	We asked: What can be done to reduce diabetes stigma?	3. Explore questions that matter
Rotation	Again, we allowed self‐selection of participants to move to a different group. Table hosts welcomed newcomers.	4. Encourage everyone's contribution
Summary of key findings	Table host reported the main points of Question 3 back to the group, and newcomers reported the main points from their previous groups' discussion. Ideas were again fed back to the event facilitator and recorded on poster paper.	5. Cross‐pollinate and connect diverse perspectives 7. Harvest and share collective discoveries
Closing talk	The lead facilitator fed back all of the ideas collected during the discussions to the group and checked for any other important points that may have been missed by recapping the notes on the poster paper. They then closed the day and thanked participants for their time.	6. Listen together for patterns, insights and deeper questions 7. Harvest and share collective discoveries

#### 2.3.3 World Café

World Café ethos proposes that communities can identify and address difficult challenges through conversation [13]. World Café is guided by seven principles: (1) setting the context; (2) creating a hospitable space; (3) exploring questions that matter; (4) encouraging everyone's contribution; (5) cross‐pollination and connection of diverse perspectives; (6) listening together for patterns, insights and deeper questions; and (7) harvesting and sharing collective discoveries [13]. Traditional focus group research is led by a moderator, creating a power dynamic whereby participants share information, but researchers often lead discussions [23]. In contrast, World Café encourages participants themselves to lead conversations and views discussion as the catalyst for action [13, 24].

Participants were divided into two small groups, and discussions were held in rounds of 20–30 min per question. After each question, some participants from each group moved to a different table where the group fed back their own discussions before the discussion continued, allowing for an enhanced exchange of knowledge. Each table was facilitated by female academic pre‐trained table hosts [25]. Some had personal and/or work‐related prior relationships with some registered attendees (*n* = 3; work colleague, student and family member). Discussions were audio‐recorded and noted on paper tablecloths, post‐its and flipcharts.

### 2.4 Data Analysis

#### 2.4.1 Survey Data

The first author, an associate professor in Qualitative Health Research experienced in several forms of pattern‐based qualitative analysis, extracted survey data into a Microsoft Excel spreadsheet. Descriptive thematic analysis [26] was used to group similar ideas and create theme labels.

To explore definitions of stigma, responses to the question ‘What does the term diabetes stigma mean to you?’ were coded inductively focusing on the explicit, or semantic [27], meaning of the data. Five labels were developed: (1) others' negative perceptions of cause (‘When someone asked what type of diabetes my husband has, then we say Type 2, they look at his stomach and nod, as if to assume its Type 2 because of his weight’.); (2) others' negative perceptions of life with diabetes (‘Judgements being made about what people can and can't do because of their diabetes’); (3) being viewed or treated negatively due to diabetes (‘People experiencing negative attitudes or treatment due to diabetes’); (4) ‘nothing’ or no response; (5) other explanation (*n* = 5, including examples of self‐stigma, lack of understanding about diabetes and some comments where the link to stigma was unclear).

Some definitions met the criteria for more than one label. When this occurred, the first author selected the label that seemed most explicitly referred to. For example, for the quote ‘To me it means people somehow see you as second rate. They seem to think you have been lazy or have had a poor diet. not in my case’, a decision was made to allocate to ‘others' negative perceptions of cause’, but this could have been labelled as ‘being viewed or treated negatively due to diabetes’. The examples of each label type were extracted and tabulated by participant group (Type 1, Type 2, Parents, Carers, Health Professionals and ‘other’). The number of instances for each label was calculated by participant group type and by experience of stigma (experienced/not experienced) and then tabulated.

Counts were converted into percentages and produced as a graphical representation, which formed the World Café preliminary findings presentation alongside label definitions and quote examples (available as Supporting Information). The labels allocated were checked by J.T., a senior lecturer in qualitative psychological research methods. Coding agreement for each label, calculated using Cohen's *κ*, ranged from 0.56 to 1 indicating moderate to perfect levels of agreement [28]. When two possible allocations could have been made, A.B. and J.T. collaboratively agreed on the final allocation. The number of instances was then recalculated, and *χ* ^2^ statistical analysis was conducted to explore the association between (1) type of diabetes and stigma meaning, and (2) whether stigma had been experienced and stigma meaning.

To explore experiences of stigma, the spreadsheet was filtered to show all cases where stigma had been experienced (*n* = 67). Responses to the question ‘Please share a little about your experience of diabetes stigma’ were extracted into a Microsoft Word table and sorted according to the participant group type. For parents and carers who indicated they had experienced stigma, any responses where the participant had indicated they knew someone else who had experienced stigma and explained these experiences in response to the question ‘Please share a little about this person's experience of diabetes stigma’ were also included. Within each group, experience statements were sorted inductively, and statement groups were given a name. This process was completed by A.B. and reviewed by J.T., and final group titles were collaboratively agreed (names and example statements for each grouping are given in Table 3).

**Table 3:** Examples of stigma experienced by respondent category.

Respondent category	Theme	Examples
Type 1	Public misperceptions about injection treatment/feeling the need to hide treatment	– I have had people with children asking to be moved away from me and my family at a restaurant as I have injected myself. – I often hide symptoms of hypos and treat hypers in private at work because it's easier to deal with it alone than to have people wrongly comment that I should be doing something differently.
	Unsolicited dietary advice	– I have had nursing colleagues comment on me putting sugar in my coffee and what I am eating for lunch because it isn't ‘diabetes appropriate’. – People don't understand the disease and appreciate that there are different types. A popular comment is always prejudging what you are eating or can and can't eat depending on ‘Type’.
	Comments about not ‘looking diabetic’	– I am constantly told I'm too skinny to be diabetic because ‘aren't all diabetics fat’.
	Misperceptions about the cause of Type 1 diabetes attributing it to diet or lifestyle	– People assume that diabetes is self‐inflicted due to diet or lifestyle. – Everyone thinks it's because I eat too much sugar that's why I have diabetes.
	Lack of understanding about the impact of type 1 diabetes on daily life/work	– How come someone else with diabetes works nights but you don't? – A lack of appreciation of how serious the disease is and its complications and that it is a 24/7 management.
	HCPs attributing all symptoms of illness to diabetes	– As an adult, doctors blame any health problem that I have on my diabetes even when it is unrelated.
	Other	– I have had colleagues get offended as I don't take a sweet or a slice of cake they have bought in. – I have had people ask if I have the ‘bad type’ of diabetes, if it's ‘the one that can make me go blind’. – Other children not wanting to sit next to me in case they caught diabetes. – I was 13 when I was diagnosed and 38 years later, I still remember being on a bus shortly after and a lady telling my mum among other negative things that I wouldn't be able to have children. Her assumption was wrong, I have two very healthy sons.
Type 2	Misperceptions about the cause (Usually public ignorance about genetic risk factors)	– I find myself explaining it's due to my dad I have diabetes, especially during job interviews. – People assume my diabetes comes from being overweight when people over my weight don't have it. I'm overweight but feel like I'm also genetically predisposed to have it too.
	Being blamed for having Type 2 diabetes	– There's also an element of people thinking ‘well you brought it on yourself’. There's a lot of ignorance about diabetes. – Told a friend I had recently been diagnosed and just said. I'm surprised you didn't have it earlier because you are fat.
	Health professional behaviours	– Health professionals making it seem self‐inflicted. Feeling judged rather than supported. – Medical professionals often assume that if my sugar levels are high, or if I have other health complications, everything is related to my diet and I should be able to fix it if I just change my diet (I eat pretty healthily) and/or lose weight. – My assigned diabetes nurse disregarded my food and exercise diaries and told me I'm type 2 diabetic and can't be doing any exercise or eating well. If I did I wouldn't be fat.
	Unsolicited advice on diet and lifestyle	– When people hear I have Type 2 they have been dismissive saying ‘Oh good you can control that with your diet can't you’. – I have many examples but one is when my husband's niece sent me a diet plan to reverse diabetes. It was kind of her to think of me, but I have a hereditary/genetic form of diabetes, and all of the dieting in the world isn't going to get rid of it.
	Others failing or refusing to make adaptations to address Type 2 diabetes needs	– In certain instances, people would serve meals that a diabetic is avoiding because they affect their immediate health and if the diabetic declines there is no other option offered. – A former colleague recently said I shouldn't be invited to a work‐related event because I would have complicated dietary requirements.
	Being treated differently at work due to Type 2 diabetes	– People on my shift would avoid working with me when I declared I had Type 2 diabetes. – Colleagues complaining that I'm getting special treatment at work.
	Other	– Life insurance doubled. Holiday insurance trebled. – Apprehensions of inadequacy in intimate relationships. – The hidden side of diabetes, for example, hypers/hypos, depression, fatigue ++. – People's assumptions and comments in media. – Not letting me decide what I need on a given day. – I feel uncomfortable when I have to explain to others while carrying my meds, especially at work or even on holidays.
Parents (Type 1)	Unsolicited advice on diet for their child	– I have experienced it on my son's behalf when he was offered fruit as a snack instead of the small slice of cake the rest of his friends were being given at school. The fruit actually had more carbs than the cake as it was such a small slice. – A child texting my daughter saying perhaps if you had a banana at break instead of a cereal bar you wouldn't have diabetes.
	Assumptions about whether diabetes is permanent/long term	– Is it for life. – Saying things to me like she will grow out of it.
	Attributions of blame on the child/parent for causing Type 1 diabetes	– I have experienced people saying to me ‘did he eat too many cakes?' Or ‘did you give him too many cakes’ with regard to my Type 1 child. It implies that he or I is to blame for his diabetes and we could have avoided it. – Received comments about how their child shouldn't eat anything containing sugar and that a poor diet must have caused their condition.
	Bullying by other children (and adults)	– My child heard another child say don't let her touch you she's diseased you will catch it. – A shop worker when buying Haribo for a hypo said to my 8‐year‐old child I thought it was just fat 50‐year‐old men who got diabetes.
	Judgements for administering injections to a child with Type 1 diabetes	– A waiter being sent over to another table as they saw me inject my daughter and complained. – I have also received nasty looks for injecting her in public.
	Child being treated differently due to diabetes	– Also partaking in school activities where food is involved (parties) can be stigmatising as she cannot just eat when she wants. – He was also somewhat excluded from a party activity at preschool once where everyone had to bring foodstuff to share, but my son had to bring his own individual plate of food and not share everyone else's.
	Other	– My daughter is unable to queue due to blood sugars and I have received nasty looks for her fast‐tracking. – People eating sweets saying I'm gonna be diabetic if I carry on. – Have been asked whether my T1 daughter has the good type of diabetes or the bad type. – Or comments that she doesn't look unhealthy. – A shop worker when buying Haribo for a hypo said to my 8‐year‐old child I thought it was just fat 50‐year‐old men who got diabetes. – Also partaking in school activities where food is involved (parties) can be stigmatising as she cannot just eat when she wants.
Carers	Attributions of blame for diabetes on the individual	– People being uneducated and not misunderstood. It can be a condition people are born with and everyone assumes it is due to their overeating or in some cases poor management. – People (health professionals) just assume it's because of his larger tummy that it's Type 2, not Type 1 diabetes. Judging him for a bad diet as such. They jump in and say, ‘ok, so it says you are diabetic, Type 2 is it?’.

To explore priorities for addressing diabetes stigma, the top three priorities posed by respondents were extracted and inductively coded by A.B. according to their intervention approach or target group. Where the strategy proposed was unclear (e.g., ‘terminology’ or ‘you are born with it’), these were excluded from the analysis. Codes were grouped to form five broader intervention categories: *General Public Intervention*: Including awareness raising, education and attitude change; *Healthcare Professional Intervention*: Including awareness raising, education, attitude and communication change; *Employer Intervention:* to raise awareness of diabetes; *Diabetes Support Intervention*: Improvements to access and psychological support; *Public Health Intervention*: Including prevention and health promotion and improving media portrayal; and *Enhanced Funding for Services and Research.* Numerical counts were performed on each of these categories and presented as a pie graph during the World Cafe to illustrate the most common top three priorities for addressing diabetes stigma (available as Supporting Information). All coding and calculations were reviewed and agreed by J.T.

#### 2.4.2 World Café Data

In World Café, the findings are produced through the discussion that takes place and the final principle and process is to harvest and share collective discoveries [13]. This feedback loop and development of themes of discussion was achieved through regular opportunities for group discussion. After each question was introduced, and discussion and rotation completed, ideas were fed back to the event facilitators and collaboratively recorded on poster paper. To facilitate this process all discussions during the World Café were audio recorded and transcribed verbatim. In addition, post‐it notes, poster paper and tablecloth notes were transcribed digitally.

Notes taken during feedback discussions were the starting point for analysis and were used by A.B. to create a structure for the findings by tabulating all issues raised (Table 4). This structure illustrates the themes identified by participants and shared during feedback discussions and therefore captures the agreed worldview.

**Table 4:** Key findings from the World Cafe discussions.

	Healthcare interaction	Public awareness	Media representation
Key issues	– People with diabetes are not seen as a whole person – Healthcare professionals lack the knowledge to support people with diabetes – People with diabetes experience judgement from healthcare professionals – Healthcare professionals are perceived as being not interested in the needs of people with diabetes	– Judgement from public – Judgement from employers and lack of adjustments made – Type 1 and Type 2 experiencing different types of stigma – Diabetes is a hidden disability – Assumptions that the person with diabetes is to blame (particularly that diabetes is due to eating too much sugar) and they should be able to help themselves	– Imagery of people with diabetes promotes stigma (e.g., people with larger bodies) – Programmes about people with diabetes perpetuate the stigma and stereotypes – There is a lack of representation of ‘real people' with diabetes in the media
Intervention needs	– Healthcare professionals need additional training on what the experience of diabetes is like – Healthcare professionals need to see the whole‐life impact of diabetes – Increase funding for diabetes support and research	– Employers need access to education, such as resource packs, to help them support employees with diabetes – Public health campaigns are needed to educate about what diabetes is really like (e.g., posters on toilet doors) – Diabetes education could be part of school curriculums	– Media representations should include images of varied ‘real people’ with diabetes – Celebrity ‘champions’ for diabetes could help break down the stigma

## 3 Results

### 3.1 Survey

#### 3.1.1 Definitions of Diabetes Stigma

Definitions were divided into four categories: being viewed or treated differently due to diabetes, others' negative perceptions of cause, others' negative perceptions of life with diabetes and others including organisational responses and self‐imposed stigma.

#### 3.1.2 Associations Between Diabetes Type, Experience of Stigma and Stigma Definitions

It was not possible to run the planned *χ* ^2^ analysis comparing all diabetes stigma definition groups due to low numbers in some groups violating the assumptions of the test (Table 5). Therefore, the smallest groups (no response/other/nothing/never heard of diabetes stigma) were combined into a single group, and two *χ* ^2^ tests were performed.

**Table 5:** Meaning of diabetes categories split by respondent type.

	Diabetes circumstance					Stigma experience	
	Type 1	Type 2	Parent	Carer	HCP	Experienced	Not experienced
Other's negative perceptions of cause	5	18	4	3	1	8	27
Other's negative perceptions of life with diabetes	7	2	2	0	1	4	8
Being viewed or treated negatively due to diabetes	16	23	1	0	5	22	26
Never heard of diabetes stigma/other/nothing/no response	4	16	3	4	1	17	6
Total	32	59	10	7	8	51	67

There was a significant association between type of diabetes (Type 1 or Type 2) and stigma meaning (*χ* ^2^ [3, *N* = 91] = 11.59, *p* = 0.009) with a moderate effect size (*w* = 0.36). Those with Type 2 diabetes were more likely to say nothing or not respond to the stigma meaning question and more likely to define stigma meaning as ‘others’ negative perception of cause' than those with Type 1. In addition, those with Type 1 diabetes were more likely to attribute stigma to others' negative perceptions of life with diabetes than those with Type 2.

There was a significant association between whether stigma had been experienced and the stigma meaning given (*χ* ^2^ [3, *N* = 118] = 15.36, *p* = 0.002) with a moderate effect size (*w* = 0.36). Those who experienced stigma were more likely than those who had not to define stigma as ‘others' negative perceptions of cause’. In addition, people who have not experienced stigma were more likely than those who have to either give no response/never heard of diabetes stigma/‘nothing’.

#### 3.1.3 Priorities for Addressing Diabetes Stigma

The most desired intervention types were public interventions including awareness raising, education and attitude change (68%). The next priorities were diabetes support interventions (15%) and HCP intervention (13%). Less common were public health intervention (6%), employer intervention (4%) and enhanced funding for services and research (4%) (available as Supporting Information S1: Figure 1).

#### 3.1.4 World Café

Findings are summarised in Table 4. The theme narrative presents these themes with illustrative quotes from the World Café.

#### 3.1.5 Issue 1: Healthcare Interaction

Healthcare interactions were often experienced negatively. Some HCPs were perceived to lack knowledge about life with diabetes and potentially hold stigma towards those with diabetes. For example, John (Type 2) described a consultation where he felt that the HCP had made presumptions about his lifestyle:

[The HCP] said ‘well, obviously you know you've got problems with your diet, you eat too much chocolate’. So, I said ‘I don't like chocolate, I don't eat chocolate’. ‘Well, what about all that sugar you have in your tea?’ I said ‘I don't have sugar in tea. I don't have sugar in coffee … I mainly eat fish; I mainly eat fruit’. And they went ‘no listen to me no, no, no, because that's, no, you must have something else’.

These experiences were believed to be grounded in a lack of understanding of the condition and what it is like to live with diabetes:

John (Type 2): Do a lot of the health professions know much about it? I don't mean that in an even remotely unkind way. I think because they're so bombarded with so much information ….

Lorna (Type 2): they're dealing with all different conditions whereas to us, we're the ones dealing with our diabetes. I've read so much about it, bought books, and looked into it. And yeah, I think we probably know more about our condition, our own particular condition, than seeing a nurse?

Although the challenge of knowing enough about all conditions was acknowledged, it was agreed that HCPs needed more education, particularly around the holistic impact of diabetes. The group want to be looked at ‘as a whole’, acknowledging not only diabetes but also other issues:

Lorna (Type 2): I just wish somebody would look at me as a whole person with the stomach issues, the diabetes, and try and do something to work out the whole thing rather than one, the diabetes people not being interested in the stomach, and the stomach people not being interested in the diabetes.

Mirza (Type 2): I would think NHS would look after me, you know, as a whole. But as you said, I learned through a hard experience that I need to look after [myself].

Participants were aware of the costs of diabetes, including the potential costs of funding to find methods to reduce stigma. Funding was identified as one potential barrier to reducing stigma:

Shane (Type 2): Only problem which comes down to is money. Always comes down to money.

John (Type 2): I was gonna say having enough people working in it, which is you just said money.

#### 3.1.6 Issue 2: Public Awareness

The group agreed that stigma resulted from a lack of awareness about diabetes, its causes and what it is like to live with the condition. This stigma was regularly experienced in day‐to‐day life. For example, Shane talked about the public response when he needed to administer medication in a restaurant:

Shane (Type 2): I was doing an injection and a waiter came over and said someone, another table, had complained, and I said ‘well tell them to come and complain to me’ … I think because people associate people doing injections and stuff with drug addicts and stuff like that, they you know, they just see someone injecting themselves, they don't know what's in there.

Participants talked about diabetes as being a ‘hidden disability’, which they felt was one of the major barriers to reducing stigma. For example, Sarah suggested that visually you would not be able to tell she had a condition, whereas with other conditions, such as cancer, it was much more visible and therefore treated differently:

Sarah (Type 1): Like everyone says, it's a hidden disability, isn't it? They see me walking around normal all of the time. I suppose it doesn't look like there's anything wrong with you. You know, if someone is in chemotherapy it's very obvious that they are ill. You know, they'll be, you know, they'll be a bit better protected [from stigma].

There was felt to be a belief, both in HCPs as mentioned in the previous issue and others, that diabetes was all about sugar consumption.

Employers were identified as a group that would benefit from education to avoid perpetuating stigma (whether intentional or not). When at work, people with diabetes could feel judged and that appropriate workplace adjustments were not being made. The group felt that resource packs for employers could be useful to address this:

Shane (Type 2): There should be resources … ‘OK, you've just been diagnosed, … Here's something for your employers. This is what your employer needs to know’.

Others talked about the importance of workplace education, for example, Mirza said:

Mirza (Type 2): I always feel if the employers can be given a course for the people that they're gonna employ, to [know to] give them a break. ‘Like, can you hang on for another hour?’ My body's telling me not. I'm type 2 diabetic. But if I'm being told, ‘wait for another hour’, I know I'm gonna pass out in the next 10 minutes. I need to go down. We're gonna take my pill. A lot of time people feel if you have [an] injection, you know they need to be given more time because they need to go ahead and inject. But you know, if you're Type 2, [they think] you can hang on for another hour, like very wrong conception about it.

Lorna similarly talked about how she felt judged by those she worked with attributing this to their lack of understanding:

Lorna (Type 2): I worked in the NHS, I was a pharmacy technician and you'd think working in the NHS in GP surgeries so even though like my boss would say ‘yes if you need to eat just leave’ but then my colleagues, if you've got a queue of people and I'm like ‘no I really need to eat now’, and they would be like, I'd say cross, they would show you that they're not happy.

The group agreed that the most needed interventions were public campaigns and changes to media representations of diabetes. They felt that this would challenge public perceptions of diabetes and address stigma, as discussed in the next theme.

#### 3.1.7 Issue 3: Media Representations

Imagery, particularly on television, was felt to create a stereotype of what living with diabetes is like, leading to negativity and attributions of the blame. Participants felt that images usually depicted a certain stereotype around diabetes, namely, people in larger bodies:

Ellie (Type 2): Everything in the [media] my gosh, … the fattest people they can find.

Lorna (Type 2): you're not just talking overweight, we're talking like literally the most overweight person you've ever seen.

Mirza (Type 2): Obese person!

Ellie talked about a perception that people believed what they saw in the media, thus perpetuating these stereotypes. Ellie went so far as to say she felt that the media portrayed being an alcoholic as more acceptable than having diabetes:

Ellie (Type 2): a certain percentage of the population believe everything what the news tells them, it's like [people with diabetes are] draining the NHS because because they don't eat well, they they they brought this on themselves and say it's like it's dirty to be diabetic, acceptable to be alcoholic but dirty to be a diabetic.

Shane also talked about his experience of watching diabetes documentaries and the stereotypes that he felt enforced, with a focus on people's eating habits, rather than the whole person:

Shane (Type 2): There is a few documentaries [on diabetes] out there, and they always seem to show like people you know. It's like, yeah, you know, ‘this is Dave, Dave likes chips’ and that's literally what you see.

The group felt that improvements could be made through targeted campaigns to educate the public. For example, Annie suggested that a simple approach used for other educational campaigns could benefit diabetes education campaigns: ‘Adverts on the back of toilets where you've got no choice but to read them’ (Annie, carer of Type 1).

The group also discussed how popular media could play a role in illustrating the real‐life experience of diabetes to help make media imagery more varied. For example, through presenting stories of real people with diabetes or perhaps engaging celebrities with diabetes in campaigns. Lorna talked about an episode of the British television programme, Strictly Come Dancing, where one of the dancers removed his shirt during the finale to show the sensor monitoring his blood sugar:

Lorna (Type 2): There's one of the Strictly Come Dancing professionals who has diabetes and nobody knew and it was his first series and he's got a [monitor] on his arm and nobody knew until he did his dance in the final he took his shirt off and everyone was like ‘what's this’ and nobody could believe that this amazing professional dancer could have diabetes and I just think more people like him, celebrities in the media.

Shane also mentioned the disbelief that people appeared to feel when the American actor Tom Hanks announced that he had diabetes:

Shane (Type 2): Like Tom Hanks when he announced a couple of years ago, he was diabetic, everyone's like, you know, it's not, it's Tom Hanks!

Challenging misrepresentation of diabetes and using different images to show that varied people have diabetes was felt to be needed in a range of areas including TV media, newspapers and written media, and even within NHS materials:

Mirza (Type 2): when you go the NHS, they show you this obese diabetic person you get so embarrassed and I keep looking left and right going is anybody looking at me, because the picture that they go to [on] the screen.

Lorna (Type 2): It would be good to show, in the Daily Mail or whatever, rather than a morbidly obese person who can't even walk if they just showed regular people with diabetes.

## 4 Discussion

This study aimed to explore stakeholder perspectives on the perceptions of diabetes stigma and strategies for addressing this. The novel World Café approach identified three main areas that participants felt impacted their experiences of stigma or had the potential to improve it: healthcare interactions, public awareness and media representations. The findings are discussed in more detail later.

From the survey, 75% of people with Type 1 diabetes and 51% of those with Type 2 reported experiencing diabetes stigma. These findings closely reflect those from the study in America conducted by Liu et al. [5] where 76% of respondents with Type 1% and 52% with Type 2 diabetes reported experiencing stigma. Similarly, the proportion of parents with children with diabetes who stated that they had experienced diabetes stigma was high in both studies (70% in our study vs. 83% in Liu et al. [5]). These findings show that diabetes stigma is experienced across Western cultures, and it appears that, consistently, those with Type 1 and parents of children with diabetes experience more stigma than those with Type 2 diabetes.

The pre‐event survey explored definitions of diabetes stigma. We found a statistically significant difference in diabetes stigma definition between those with Type 1 and those with Type 2 diabetes. Participants with Type 2 diabetes were more likely to define stigma as others' negative perception of cause, whereas participants with Type 1 were more likely to define stigma as others' negative perception of life with diabetes. These findings reflect those in other studies [29, 30] where stigma in people with Type 2 diabetes is linked to blame and shame for causing their condition. However, in contrast to other studies, we did not find that participants with Type 1 diabetes reported stigma by association with Type 2 diabetes [31].

The World Café enabled the group to review and engage with the findings discussed earlier and explore these in more depth. This resulted in detailed discussions and rich data, with the participants raising important issues regarding how diabetes is portrayed by others, sharing their experiences of attitudes of others towards them and discussing collaboratively potential solutions for raising the profile of diabetes to reduce stigma. Sources of stigma came from individuals, HCPs, media, the public and employers, supporting the findings from other studies [29, 30, 31].

HCPs were frequently discussed as sources of stigma, with some perceived as making assumptions (e.g., that they had diabetes because they had eaten too much sugar) and others seemingly having little knowledge about the condition. These experiences align with research evidence that suggests that many HCPs hold stigma towards patients with diabetes [32, 33, 34] with one study suggesting as many as one‐third may view patients with Type 2 diabetes as lazy and lacking motivation [35]. Participants reported feeling judged and not seen as an individual, suggesting a loss of trust in the knowledge of the HCPs that they had appointments with, concerningly these perceived experiences of stigma in healthcare settings can increase the risk of depression and other negative health outcomes [34]. Stigma experiences reported related to features of the condition and its management such as people's eating habits, needle use and the misconceptions that Type 2 diabetes was less severe than Type 1 diabetes. There were also perceptions that diabetes ‘costs money’ and that it is a burden on the healthcare system, again mirroring those findings from Liu et al. [5]. These experiences made people feel judged and misunderstood, and they contributed to the development of negative stereotypes about people with diabetes [10], highlighting a clear need for interventions to educate HCPs about diabetes stigma as recommended by the participants.

The participants discussed the importance of education for reducing diabetes stigma, and of changing the perception of diabetes in the media, for example, educating people that it is a medical condition that affects the pancreas, rather than a condition that is associated with a poor lifestyle. Position statements from international diabetes organisations (including Diabetes Australia [36], the American Association of Diabetes Educators, the American Diabetes Association [37] and the English Advisory Group [38]) have already started to make important changes by providing guidance and education on the language used in discussing diabetes and diabetes care. However, further work is needed to move this forward and change the way diabetes is discussed within both the healthcare system and the media and public discourse.

Participants also stated that they would like to see resources for employers, which is interesting as resources like these are freely available from some diabetes organisations (e.g., Diabetes UK). Therefore, it seems that some of the participants and employers were not aware of these resources, and more signposting would be useful, particularly at key points such as at diagnosis. With the increasing focus and importance placed on patient and public involvement in improving the quality of health care [39], co‐designed and co‐produced resources, involving people with diabetes and practitioners would be helpful in the future to help educate and inform those who employ or care for people with diabetes, as well as the public.

### 4.1 Limitations

The format of a World Café involves discussion and sharing of ideas with a wide range of people and concerns about the confidentiality of such discussion may have been a barrier to contribution for some participants [25]. This may be why two participants on the day chose to listen to discussions but did not verbally contribute. In addition, all World Café participants self‐selected for the event and were motivated to be involved. Those who attended were mostly participants with Type 2 diabetes (*n* = 7), which meant that representations of those who were carers, HCPs, parents or people with Type 1 diabetes were under‐represented. However, this was in some way mitigated by the inclusion of the survey that provided perspectives from a much wider range of stakeholders, and by sharing these with the group before discussions these perspectives contributed to the development of the overall worldview.

## 5 Conclusions

The findings from this study supported previous research by demonstrating that diabetes stigma is experienced by people with Type 1 and Type 2 diabetes. Sources of stigma came from individuals, HCPs, media, the public and employers. The World Café methodology proved to be an excellent method for sharing knowledge and experiences among stakeholders and identifying stakeholder priorities for future strategies to bring about change. Results indicate that further work is needed to change the way diabetes is discussed both within the healthcare system as well as within the media and public discourse.

## Author Contributions

**Amy Elizabeth Burton:** conceptualisation, methodology, formal analysis, investigation, data curation, writing–original draft, writing–review and editing. **Alison Owen:** formal analysis, investigation, data curation, writing–original draft, writing–review and editing. **Jennifer Taylor:** formal analysis, investigation, data curation, writing–original draft, writing–review and editing. **Sarah Elizabeth Dean:** formal analysis, investigation, data curation, writing–original draft, writing–review and editing. **Rachel Povey:** conceptualisation, investigation, writing–original draft, writing–review and editing.

## Ethics Statement

Ethical approval was obtained from the Staffordshire University Health, Science and Wellbeing Ethics Committee on 15 May 2023 (number SU_22_284).

## Conflicts of Interest

The authors declare no conflicts of interest.

## Supporting information

Supporting information.

## Data Availability

The data that support the findings of this study are available from the corresponding author (A.B.) upon reasonable request. The data are not publicly available due to containing information that could compromise the privacy of research participants.

## References

[1] K. L. Ong , L. K. Stafford , S. A. McLaughlin , et al., “Global, Regional, and National Burden of Diabetes From 1990 to 2021, with Projections of Prevalence to 2050: A Systematic Analysis for the Global Burden of Disease Study 2021,” Lancet 402, no. 10397 (2023): 203–234, 10.1016/S0140-6736(23)01301-6. 37356446 PMC10364581

[2] Diabetes UK, “How Many People in the UK Have Diabetes?,” 2024, http://www.diabetes.org.uk/about-us/about-the-charity/our-strategy/statistics.

[3] World Health Organization, “Diabetes,” 2024, http://www.who.int/health-topics/diabetes.

[4] Centres for Disease Control and Prevention, “Diabetes Stigma: Learn About It, Recognize It, Reduce It,” accessed November 20, 2023, http://www.cdc.gov/diabetes/library/features/diabetes_stigma.html.

[5] N. F. Liu , A. S. Brown , A. E. Folias , et al., “Stigma in People With Type 1 or Type 2 Diabetes,” Clinical Diabetes 35, no. 1 (2017): 27–34, 10.2337/cd16-0020. 28144043 PMC5241772

[6] J. L. Browne , A. Ventura , K. Mosely , and J. Speight , “‘I'm Not a Druggie, I'm Just a Diabetic’: A Qualitative Study of Stigma From the Perspective of Adults With Type 1 Diabetes,” BMJ Open 4, no. 7 (2014): e005625, 10.1136/bmjopen-2014-005625. PMC4120421 25056982

[7] J. A. Nettleton , A. E. Burton , and R. C. Povey , “No‐One Realises What We Go Through As Type 1s”: A Qualitative Photo‐Elicitation Study on Coping With Diabetes,” Diabetes Research and Clinical Practice 187 (2022): 109876. 35439539 10.1016/j.diabres.2022.109876

[8] J. L. Browne , A. Ventura , K. Mosely , and J. Speight , “I Call It the Blame and Shame Disease’: A Qualitative Study about Perceptions of Social Stigma Surrounding Type 2 Diabetes,” BMJ Open 3, no. 11 (2013): e003384, 10.1136/bmjopen-2013-003384. PMC3840338 24247325

[9] The Lancet Diabetes Endocrinology , “Diabetes Stigma and Discrimination: Finding the Right Words,” Lancet. Diabetes & Endocrinology. 6, no. 9 (2018): 673, 10.1016/S2213-8587(18)30235-3. 30143183

[10] J. Schabert , J. L. Browne , K. Mosely , and J. Speight , “Social Stigma in Diabetes: A Framework to Understand a Growing Problem for an Increasing Epidemic,” Patient ‐ Patient‐Centered Outcomes Research 6, no. 1 (2013): 1–10, 10.1007/s40271-012-0001-0. 23322536

[11] F. Cornish , N. Breton , U. Moreno‐Tabarez , et al., “Participatory Action Research,” Nature Reviews Methods Primers 3, no. 1 (2023): 34, 10.1038/s43586-023-00214-1.

[12] J. Jagosh , A. C. Macaulay , P. Pluye , et al., “Uncovering the Benefits of Participatory Research: Implications of a Realist Review for Health Research and Practice,” Milbank Quarterly 90, no. 2 (2012): 311–346, 10.1111/j.1468-0009.2012.00665.x. 22709390 PMC3460206

[13] J. Brown and D. Isaacs , World Cafe Community , The World Café: Shaping Our Futures Through Conversations That Matter (Oakland, USA: Berrett‐Koehler Publishers, 2005), http://hesge.scholarvox.com/book/88806898.

[14] K. Löhr , M. Weinhardt , and S. Sieber , “The ‘World Café’ as a Participatory Method for Collecting Qualitative Data,” International Journal of Qualitative Methods 19 (2020): 160940692091697, 10.1177/1609406920916976.

[15] L. Khong , C. Bulsara , K. D. Hill , and A. M. Hill , “How Older Adults Would Like Falls Prevention Information Delivered: Fresh Insights From a World Café Forum,” Ageing and Society 37, no. 6 (2017): 1179–1196, 10.1017/S0144686X16000192.

[16] V. Recchia , A. Dodaro , E. De Marco , and A. Zizza , “A Critical Look to Community Wisdom: Applying the World Café Method to Health Promotion and Prevention,” International Journal of Health Planning and Management 37 (2022): 220–242, 10.1002/hpm.3594. 36411997

[17] M. Banfield , A. Gulliver , and A. R. Morse , “Virtual World Café Method for Identifying Mental Health Research Priorities: Methodological Case Study,” International Journal of Environmental Research and Public Health 19, no. 1 (2021): 291, 10.3390/ijerph19010291. 35010550 PMC8744911

[18] A. J. Clements , A. Sharples , and G. Kinman , “Identifying Well‐Being Challenges and Solutions in the Police Service: A World Café Approach,” Police Journal: Theory, Practice and Principles 94, no. 2 (2021): 81–101, 10.1177/0032258X19898723.

[19] J. Woodhall‐Melnik , S. Hamilton‐Wright , E. Hamilton‐Wright , S. J. T. Guilcher , and F. I. Matheson , “Getting Help: Findings From Two World Cafés With Youth Who Experience Homelessness,” Canadian Journal of Family and Youth/Le Journal Canadien de Famille et de la Jeunesse 14, no. 3 (2022): 52–77, 10.29173/cjfy29797.

[20] M. D. Fetters , L. A. Curry , and J. W. Creswell , “Achieving Integration in Mixed Methods Designs—Principles and Practices,” Health Services Research 48, no. 6 (2013): 2134–2156, 10.1111/1475-6773.12117. 24279835 PMC4097839

[21] The data analysis for this paper was generated using Qualtrics software Copyright © [2022] Qualtrics, Qualtrics and all other Qualtrics product or service names are registered trademarks or trademarks of Qualtrics, Provo, UT, USA, https://www.qualtrics.com.

[22] V. Braun and V. Clarke , Successful Qualitative Research: A Practical Guide for Beginners (London, UK: Sage, 2013).

[23] J. Kitzinger , “The Methodology of Focus Groups: the Importance of Interaction Between Research Participants,” Sociology of Health & Illness 16, no. 1 (1994): 103–121.

[24] K. Löhr , M. Weinhardt , and S. Sieber , “The ‘World Café’ as a Participatory Method for Collecting Qualitative Data,” International Journal of Qualitative Methods 19 (2020): 1609406920916976, 10.1177/1609406920916976.

[25] S. Page , “People Get Killed Cause of There [Their] Skin. It Cannot Be Stopped’: A Midlands Case Study Considering Experiences of Racism Amongst Pupils in UK Secondary Schools and the Community,” British Journal of Community Justice 16, no. 1 (2020): 64.

[26] R. E. Boyatzis , Transforming Qualitative Information: Thematic Analysis and Code Development (London, UK: Sage, 1998).

[27] V. Clarke and V. Braun , Thematic Analysis: A Practical Guide (London, UK: Sage Publications, 2021).

[28] C. Robson , Real World Research: A Resource for Social Scientists and Practitioner‐Researchers (Oxford, UK: Blackwell, 1993).

[29] J. Schabert , J. L. Browne , K. Mosely , and J. Speight , “Social Stigma in Diabetes: A Framework to Understand a Growing Problem for an Increasing Epidemic,” Patient ‐ Patient‐Centered Outcomes Research 6, no. 1 (2013): 1–10, 10.1007/s40271-012-0001-0. 23322536

[30] J. L. Browne , A. Ventura , K. Mosely , and J. Speight , “I Call It the Blame and Shame Disease’: A Qualitative Study About Perceptions of Social Stigma Surrounding Type 2 Diabetes,” BMJ Open 3, no. 11 (2013): e003384‐003384, 10.1136/bmjopen-2013-003384. PMC3840338 24247325

[31] J. L. Browne , A. Ventura , K. Mosely , and J. Speight , “I'm Not a Druggie, I'm Just a Diabetic’: A Qualitative Study of Stigma From the Perspective of Adults With Type 1 Diabetes,” BMJ Open 4, no. 7 (2014): e005625‐005625, 10.1136/bmjopen-2014-005625. PMC4120421 25056982

[32] R. Embick , M. Jackson , and R. Stewart , “The Impact of Stigma on the Management of Type 1 Diabetes: A Systematic Review,” Diabetic Medicine 41, no. 4 (2024): e15299, 10.1111/dme.15299. 38361327

[33] J. K. Dickinson , “The Experience of Diabetes‐Related Language in Diabetes Care,” Diabetes Spectrum 31, no. 1 (2018): 58–64, 10.2337/ds16-0082. 29456427 PMC5813309

[34] H. Budhwani , P. De , and R. Sun , “Perceived Stigma in Health Care Settings Mediates the Relationships Between Depression, Diabetes, and Hypertension,” Population Health Management 25, no. 2 (2022): 164–171, 10.1089/pop.2021.0268. 35442794 PMC9058871

[35] B. L. Bennett and R. M. Puhl , “Diabetes Stigma and Weight Stigma Among Physicians Treating Type 2 Diabetes: Overlapping Patterns of Bias,” Diabetes Research and Clinical Practice 202 (2023): 110827, 10.1016/j.diabres.2023.110827. 37451627

[36] J. Speight., T. C. Skinner , T. Dunning , et al., “Our Language Matters: Improving Communication With and About People With Diabetes. A Position Statement by Diabetes Australia,” Diabetes Research and Clinical Practice 173 (2021): 108655, 10.1016/j.diabres.2021.108655. 33422586

[37] J. K. Dickinson , S. J. Guzman , M. D. Maryniuk , et al., “The Use of Language in Diabetes Care and Education,” Diabetes Care 40, no. 12 (2017): 1790–1799, 10.2337/dci17-0041. 29042412

[38] A. Cooper , N. Kanumilli , J. Hill , et al., “Language Matters. Addressing the Use of Language in the Care of People With Diabetes: Position Statement of the English Advisory Group,” Diabetic Medicine 35, no. 12 (2018): 1630–1634, 10.1111/dme.13705. 29888553

[39] S. Fereday and K. Rezel , Patient and Public Involvement in Quality Improvement (Health Quality Improvement Partnership, 2017).

