# Supplementary material for: A World Café Approach to Exploring Perspectives on Diabetes Stigma in the United Kingdom
Source: Health Expect. 2024 Sep 6;27(5):e70023. doi: 10.1111/hex.70023 (PMC11377845; doi:10.1111/hex.70023)
Supplement: Supplementary file 1 — Supporting information. [file HEX-27-e70023-s001.docx]

# Materials included in the World Café presentation

These slides were presented to the group attending the World Café to set context for the discussions that followed and to ensure discussions were grounded in an understanding of responses from the full survey sample.


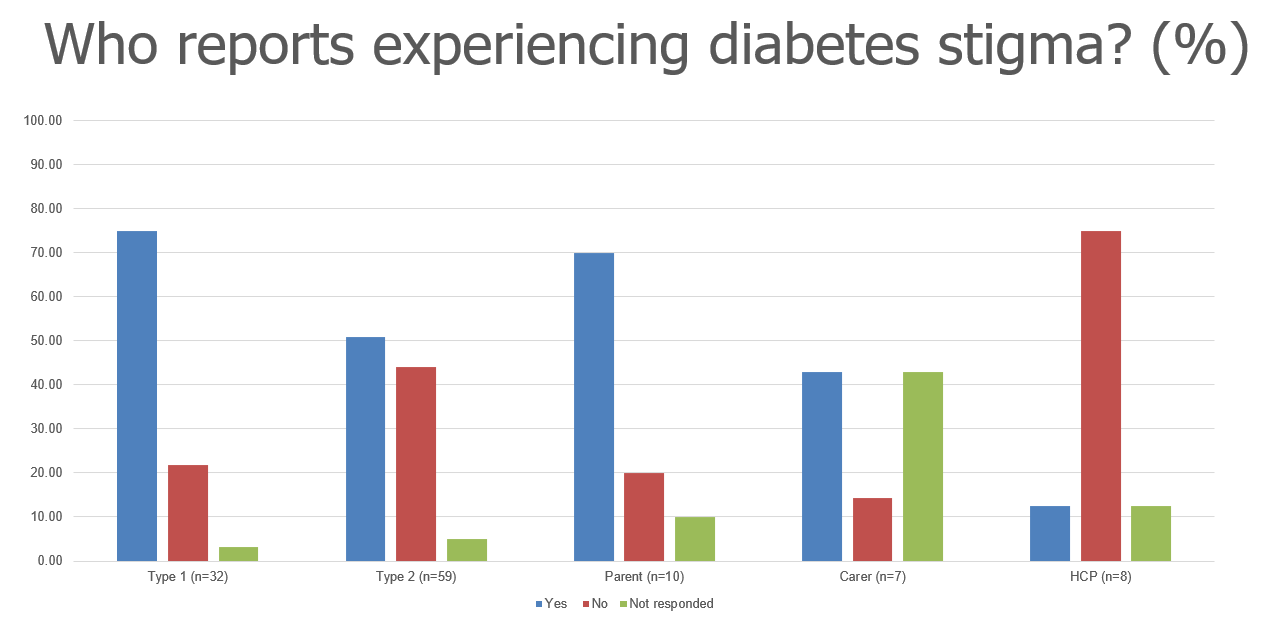


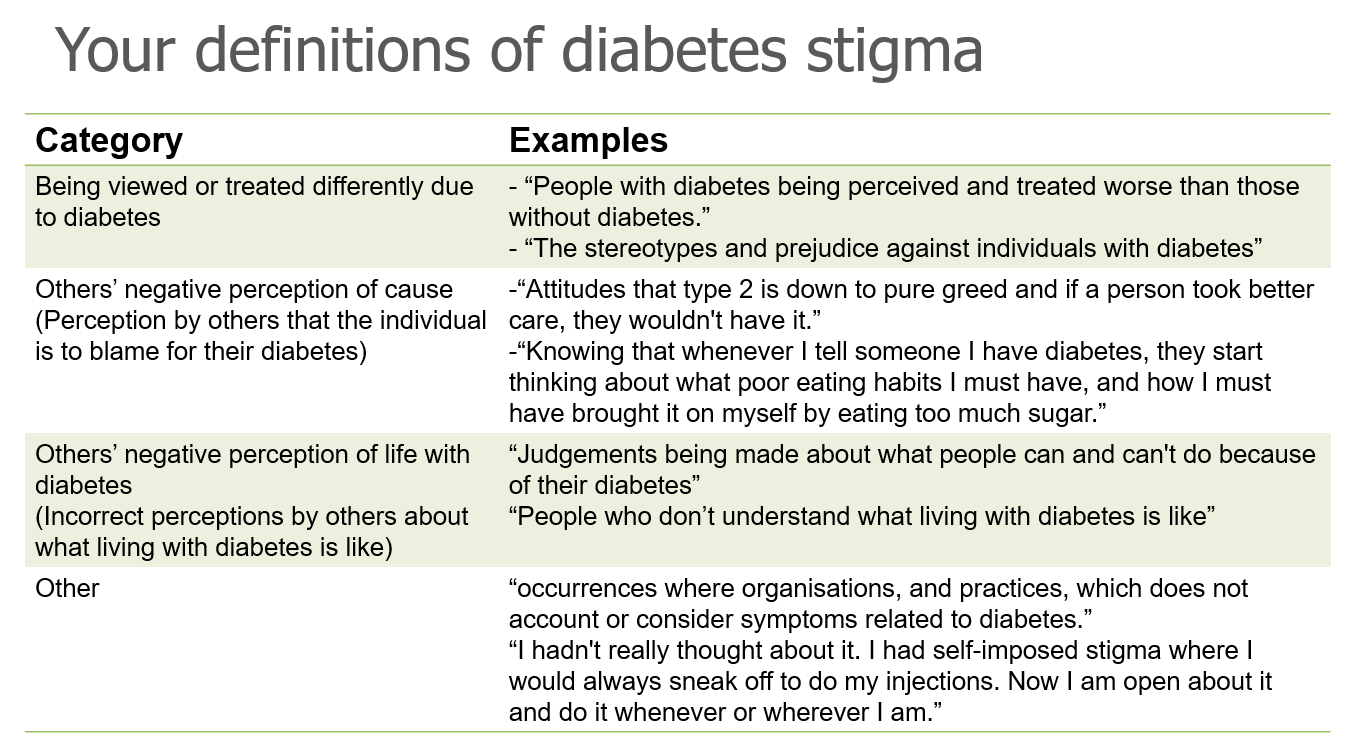


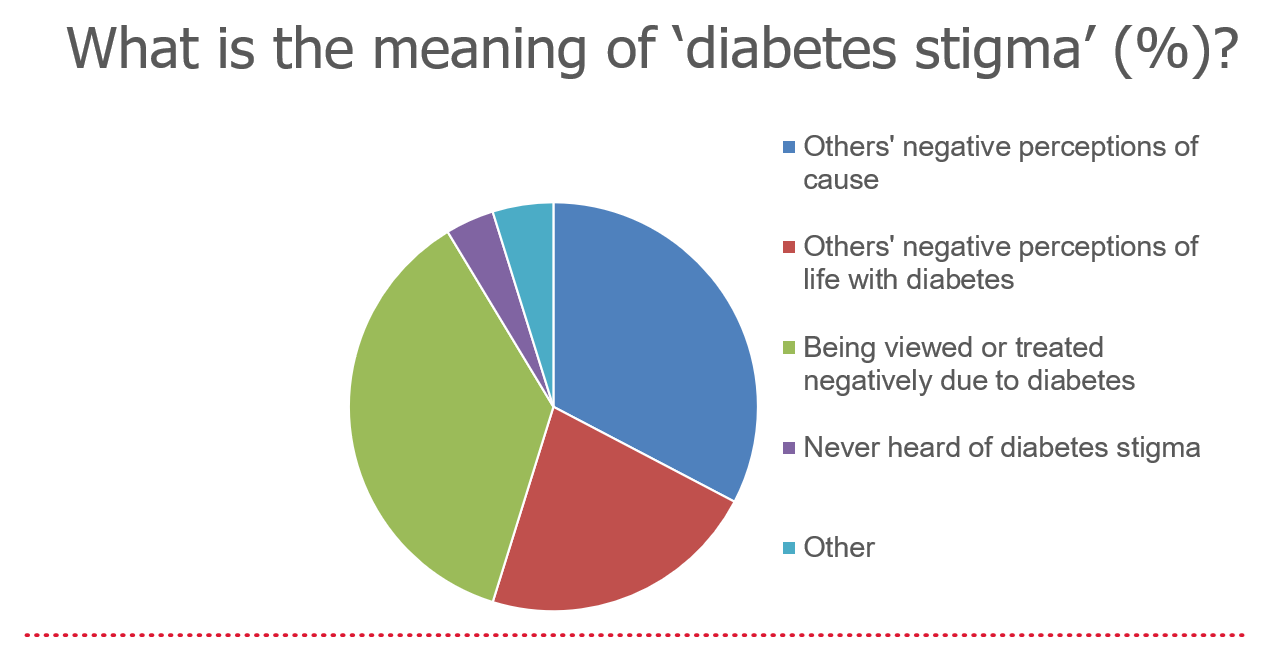


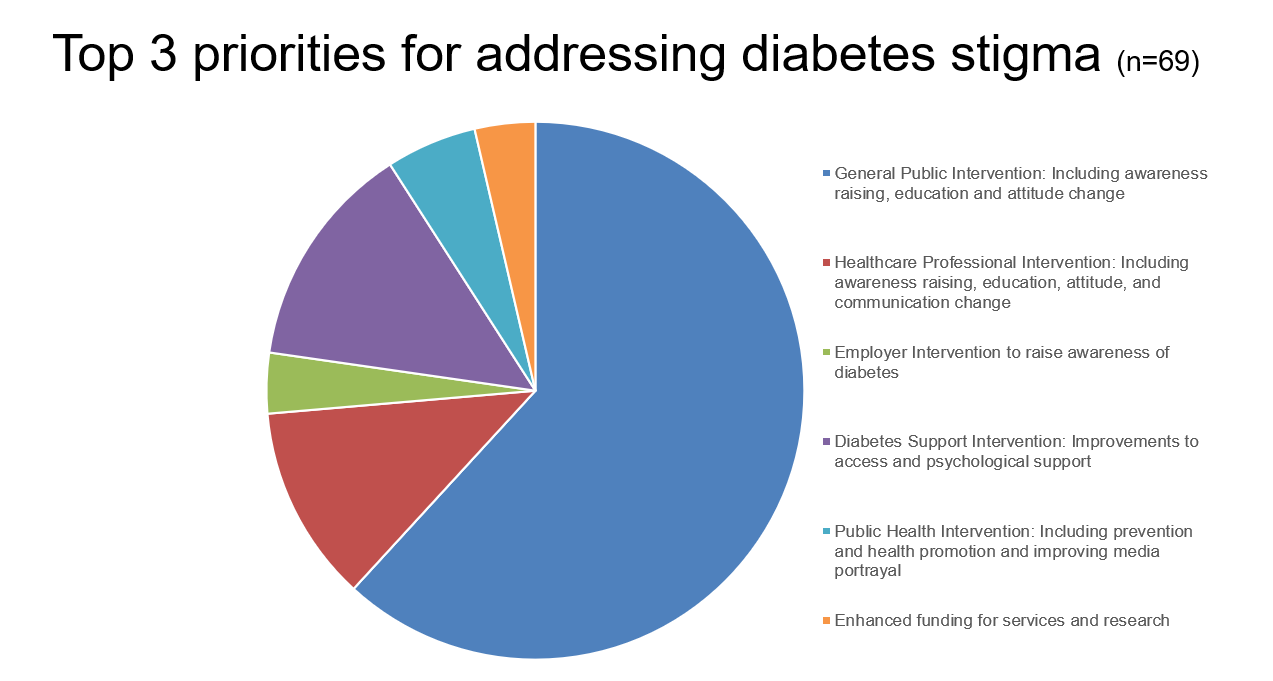


Figure 1: Percentage of top 3 priorities for diabetes stigma intervention in each category
